# BIM deletion polymorphisms in Hispanic patients with non-small cell lung cancer carriers of EGFR mutations

**DOI:** 10.18632/oncotarget.12112

**Published:** 2016-09-19

**Authors:** Andrés F. Cardona, Leonardo Rojas, Beatriz Wills, Oscar Arrieta, Hernán Carranza, Carlos Vargas, Jorge Otero, Luis Corrales-Rodriguez, Claudio Martín, Noemí Reguart, Pilar Archila, July Rodríguez, Mauricio Cuello, Carlos Ortíz, Sandra Franco, Christian Rolfo, Rafael Rosell, on behalf of the CLICaP

**Affiliations:** ^1^ Clinical and Traslational Oncology Group, Institute of Oncology, Clínica del Country, Bogotá, Colombia; ^2^ Foundation for Clinical and Applied Cancer Research (FICMAC), Bogotá, Colombia; ^3^ Clinical and Traslational Research Department, Faculty of Medicine, Universidad el Bosque, Bogotá, Colombia; ^4^ Clinical Oncology Department, Centro Javeriano de Oncología, Hospital Universitario San Ignacio, Bogotá, Colombia; ^5^ Faculty of Medicine, Pontificia Universidad Javeriana, Bogotá, Colombia; ^6^ Thoracic Oncology Unit, Instituto Nacional de Cancerología (INCan), México City, México; ^7^ Medical Oncology Department, Hospital San Juan de Dios, San José, Costa Rica; ^8^ Thoracic Oncology Unit, Alexander Fleming Institute, Buenos Aires, Argentina; ^9^ Medical Oncology Department, Hospital Clínic, Barcelona, Spain; ^10^ Clinical Oncology Department, Hospital de Clínicas-UdeLAR, Montevideo, Uruguay; ^11^ Early Clinical Trials Unit, Oncology Department, Antwerp University Hospital and Center for Oncological Research (CORE), Antwerp University, Edegem, Belgium; ^12^ Medical Oncology Department, Catalan Institute of Oncology, Hospital Germans Trias i Pujol, Badalona, Barcelona, Spain

**Keywords:** non-small cell lung cancer, BIM deletion, EGFR mutation, survival

## Abstract

**Background:**

Germline alterations in the proapoptotic protein Bcl-2-like 11 (BIM) can have a crucial role in diverse tumors. To determine the clinical utility of detecting BIM deletion polymorphisms (par4226 bp/ par363 bp) in EGFR positive non-small-cell lung cancer (NSCLC) we examined the outcomes of patients with and without BIM alterations.

**Results:**

BIM deletion was present in 14 patients (15.7%). There were no significant differences between patients with and without BIM*-del* in clinical characteristics or EGFR mutation type; however, those with BIM*-del* had a worse overall response rate (ORR) to erlotinib (42.9% vs. 73.3% in patients without BIM*-del*; p=0.024) as well as a significantly shorter progression-free survival (PFS) (10.8 BIM*-del+* vs. 21.7 months for patients without BIM*-del*; p=0.029) and overall survival (OS) (15.5 BIM*-del+* vs. 34.0 months for patients without BIM*-del*; p=0.035). Multivariate Cox regression analysis showed that BIM*-del+* was an independent indicator of shorter PFS (HR 3.0; 95%CI 1.2-7.6; p=0.01) and OS (HR 3.4; 95%CI 1.4-8.3; p=0.006).

**Methods:**

We studied 89 NSCLC Hispanic patients with EGFR mutation who were treated with erlotinib between January 2009 and November 2014. BIM deletion polymorphisms (BIM*-del*) was analyzed by PCR in formalin-fixed paraffin-embedded (FFPE) tissues of tumor biopsies. We retrospectively analyzed clinical characteristics, response rate, toxicity, and outcomes among patients with and without BIM-*del*.

**Conclusions:**

The incidence of BIM*-del* found in Hispanic patients is similar to that previously described in Asia. This alteration is associated with a poor clinical response to erlotinib and represents an independent prognostic factor for patients who had NSCLC with an EGFR mutation.

## INTRODUCTION

Lung cancer is the leading cause of cancer related death in the developed countries and in Latin America, and non–small-cell lung cancer (NSCLC) accounts for most cases [1, 2]. Activating mutations in the epidermal growth factor receptor (*EGFR*) as a therapeutic target for NSCLC has changed the course of the disease [3]. The frequency of *EGFR* mutations vary according to the population; in Caucasians EGFR mutations occurs in 10 to 15%, whereas in East Asia and Latin America these are more frequent occurring in 30 to 50% of lung adenocarcinoma patients [4–6]. EGFR tyrosine kinase inhibitors (TKIs), such as gefitinib, erlotinib, and afatinib, are widely used to treat advanced NSCLC harboring an EGFR mutation. Such drugs have improved the progression free survival (PFS), overall survival (OS) and quality of life compared with first line platinum-based doublet chemotherapy [7–10]. However, drug resistance invariably emerged and most patients develop recurrence within 10 to 16 months after initial EGFR-TKI treatment (acquired resistance) [11]. Several mechanisms of secondary resistance have been revealed, including: EGFR T790M mutation (the most frequent), mesenchymal-epithelial transition, *MET* amplification, phosphatidylinositol-4-5-bisphosphate 3-kinase mutations (*PI3K*) and small-cell lung cancer transformation [12–15]. Nevertheless, around 30% of patients with EGFR-activating mutations do not show objective response (OR) to EGFR TKIs (primary resistance) [7, 8]. The mechanisms and characteristics of primary resistance are less known and none of these explain the majority of cases. Some of mechanisms of primary resistance include: v-Ki-ras2 Kirsten rat sarcoma viral oncogene homolog (*KRAS*) mutations, de novo *MET* amplification, and phosphatase and tensin-homolog (*PTEN*) loss [16–19]. An interesting mechanism related with germline polymorphisms is proapoptotic protein Bcl-2-like 11 (BIM) which has been described and could potentially explain primary resistance to EGFR TKIs [20].

BIM is a member of the B-cell CLL/Lymphoma 2 (Bcl-2) family of proteins and has been related with apoptosis modulation triggered by EGFR-TKIs [21–23]. BIM deletion polymorphisms (BIM*-del*) consist of intronic deletion polymorphisms in the gene. These polymorphisms switched BIM splicing from exon 4 to exon 3, which resulted in expression of BIM isoforms lacking the proapoptotic Bcl-2-homology domain 3 (BH3) [20]. These germline alterations could have a crucial role in determining how a tumor responds to EGFR-TKIs; however, few studies (none from Latin America) have examined the clinical usefulness of detecting BIM deletion polymorphisms and its relation with clinical characteristics in EGFR positive NSCLC. To determine the usefulness of detecting BIM*-del* in patients with EGFR mutation-positive NSCLC, we examined the outcomes of Hispanic patients with and without BIM alterations.

## RESULTS

### Demographic and clinicopathologic characteristics

The characteristics of the patients included in the study are summarized in Table 1. As expected in EGFR mutated patients, adenocarcinoma histology and non-smokers were both frequent characteristics. EGFR common mutations were present in the majority of patients (84/89 patients) including deletion of exon 19 (46 patients) and L858R (38 patients). BIM*-del* was present in 14 patients (15.7%). There were no significant differences between patients with and without BIM*-del* regarding clinical characteristics or type of EGFR mutation, but a difference was obtained with previous tobacco exposure (p = 0.04) (Table 2).

**Table 1:** Patient characteristics according to Bcl-2-Like Protein 11 (BIM) deletion polymorphism

Variable	N = 89 (%)	*BIM-del*+ N=14 (%)	BIM *del*- N=75 (%)	P-value
**Gender**				
Female	62 (69.7)	9 (64.3)	53 (70.7)	0.06
Male	27 (30.3)	5 (35.7)	22 (29.3)	
**Age, mean**	59.4 (+/− 14.3)	52.6 (+/− 13.7)	60.8 (+/− 11.8)	0.07
>60 years	50 (56.2)	5 (35.8)	45 (60.0)	
<60 years	39 (43.8)	9 (64.2)	30 (40.0)	
**ECOG**				
0	11 (12.4)	2 (14.3)	9 (12.0)	0.54
1	44 (49.4)	5 (35.7)	39 (52.0)	
2	31 (34.8)	7 (50.0)	24 (32.0)	
3	3 (3.4)	-	3 (4.0)	
ND	-	-	-	
**Stage**				
IIIA	1 (1.1)	-	1 (1.3)	0.78
IIIB	4 (4.5)	-	4 (5.3)	
IV	84 (94.4)	14 (100.0)	70 (93.3)	
**Histology**				
Adenocarcinoma	87 (97.8)	14 (100.0)	73 (96.8)	0.63
LCC	1 (1.1)		1 (1.6)	
NOS/Adenosquamous	1 (1.1)		1 (1.6)	
**Histologic pattern (adenocarcinoma)**				
Lepidic	9 (10.1)	2 (14.3)	7 (9.3)	0.53
Acinar	10 (11.2)	-	10 (13.3)	
Papillary	17 (19.1)	2 (14.3)	15 (20.0)	
Micropapillary	17 (19.1)	3 (21.4)	14 (18.7)	
Solid	4 (4.5)	-	4 (5.3)	
ND	32 (36.0)	7 (50.0)	25 (33.3)	
**Smoking history**				
Never	50 (56.2)	11 (78.6)	39 (52.0)	0.04
Former/Current	37 (41.6)	3 (21.4)	34 (45.3)	
ND	2 (2.2)		2 (2.7)	
**Pleuro/pulmonary metastases**				
Yes	44 (49.4)	5 (35.7)	39 (52.0)	0.60
No	40 (44.9)	9 (64.3)	31 (41.3)	
ND	5 (5.6)	-	5 (6.7)	
**CNS metastases**				
Yes	34 (38.2)	6 (42.9)	28 (37.3)	0.58
No	48 (53.9)	8 (57.1)	40 (53.3)	
ND	7 (7.9)	-	7 (9.3)	
**Liver metastases**				
Yes	33 (37.1)	7 (50.0)	26 (34.7)	0.72
No	50 (56.2)	7 (50.0)	43 (57.3)	
ND	6 (6.7)	-	6 (8.0)	
**Bone metastases**				
Yes	39 (43.8)	6 (42.9)	33 (44.0)	0.65
No	48 (53.9)	8 (57.1)	40 (53.3)	
ND	2 (2.2)	-	2 (2.7)	
**Lymph node metastases**				
Yes	43 (48.4)	10 (71.4)	33 (44.0)	0.60
No	46 (51.6)	4 (28,6)	42 (56.0)	
**Weight loss**				
Yes	45 (50.6)	7 (50.0)	38 (50.7)	0.78
No	40 (44.9)	6 (42.9)	34 (45.3)	
ND	4 (4.5)	1 (7.1)	3 (4.0)	

LCC: Large Cell Carcinoma; NOS: Not Otherwise Specified

**Table 2:** EGFR and BIM distribution

Variable	N=89 (%)
**Type of EGFR mutation**	
Common	84 (94.4)
Uncommon	5 (5.6)
**EGFR subgroup**	
*Del19* (12 pb)	46 (50.7)
L858R	38 (42.6)
G719X	5 (6.7)
**BIM global**	
Positive	14 (15.7)
Negative	75 (84.3)
**BCL2-like 11 par 4226 bp**	
Negative	78 (87.6)
Positive	11 (12.4)
**BCL2-like 11 par 363 bp**	
Negative	79 (88.8)
Positive	10 (11.2)

### Response to TKI therapy and survival

**Figure 1:**
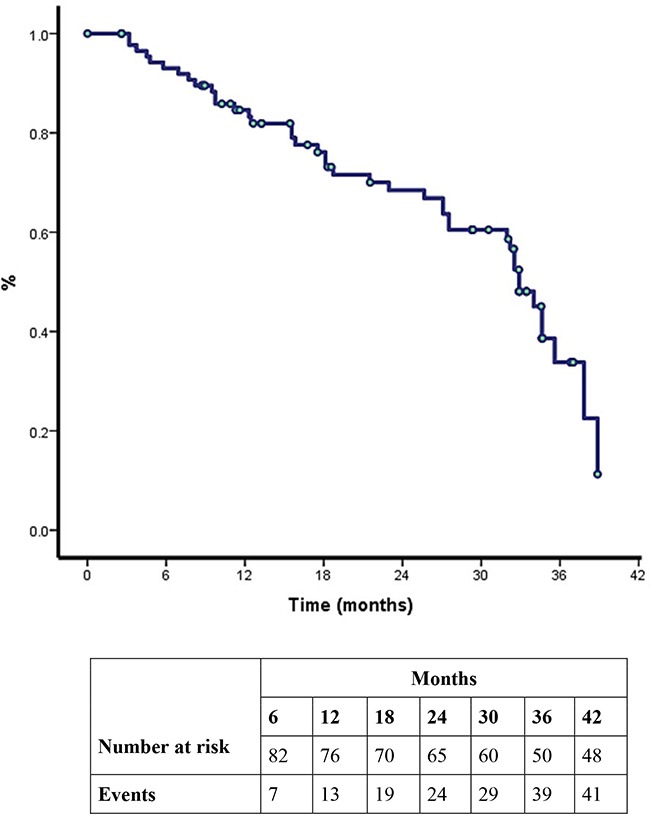
**A.** Kaplan-Meier curve for overall survival (OS) after epidermal growth factor receptor (EGFR)-tyrosine kinase inhibitor treatment. **B.** Progression free survival.

**Figure 2:**
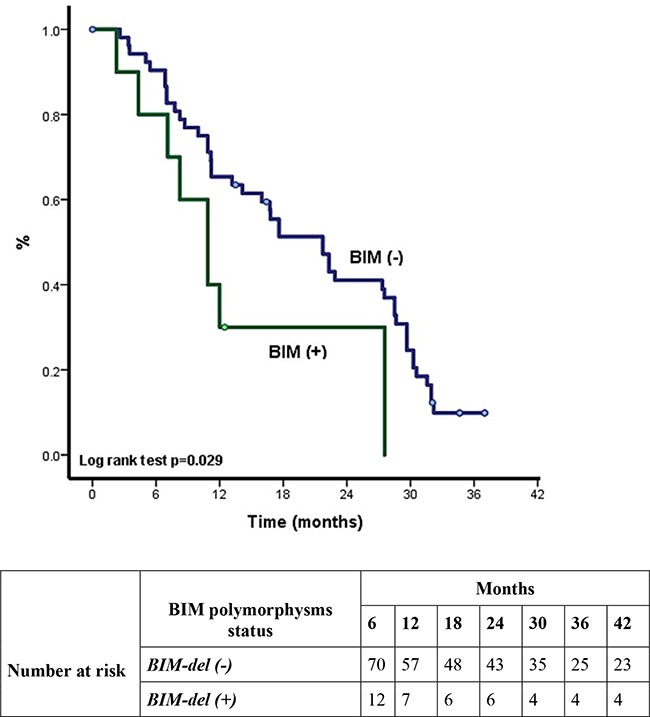
**A.** Overall survival in EGFR+ according to BIM status. **B.** Progression free survival in EGFR+ according to BIM-*del* status.

There was a significant difference in ORR between patients with and without BIM*-del*. Patients who were BIM*-del*+ had a worse ORR to erlotinib compared to patients with a BIM *del-* (42.9% vs. 73.3%; p=0.024) (Table 3). There was no difference in ORR to chemotherapy between BIM*-del+* and BIM *del-* populations (Table 3). Overall survival (OS) was 32.9 months (95% CI 31.1-34.6) and overall PFS was 19.5 months (95% CI 9.7-25.4) (Figure 1A and 1B). Patients with BIM*-del+* had a significantly shorter PFS (10.8 vs. 21.7 months for those patients without BIM*-del*; p=0.029) (Figure 2A) and detrimental OS (15.5 vs. 34.0 months for patients without BIM*-del*; p=0.035) (Figure 2B). Multivariate Cox regression analysis showed that BIM*-del* was an independent indicator of shorter PFS (HR 3.0; 95%CI 1.2-7.6; p=0.01) and OS (HR 3.4; 95%CI 1.4-8.3; p=0.006) (Table 3).

**Table 3:** Response rate in EGFR+ according to *BIM-del* status

Response rate	*BIM-del+* N=14 (%)	*BIM del-* N=75 (%)	P
**Response to TKIs**			
Yes	5 (35.7)	55 (73.3)	0.002
No	9 (64.3)	20 (26.7)	
**Response to chemotherapy**			
Yes	4 (28.6)	24 (32.0)	0.67
No	5 (35.7)	23 (30.7)	
ND	5 (35.7)	28 (37.3)	

### Toxicity

Thirty-eight (42.6%) patients suffered grade 3 or 4 adverse event. Most patients experienced rash (36%), fatigue (30%), diarrhea (16%) and anorexia (10%), but no unexpected serious adverse reactions were reported. Major toxicity was not influenced by BIM*-del* (p=0.68).

## DISCUSSION

Several studies have demonstrated that BIM deletion polymorphism is related with response to EGFR TKIs in NSCLC [20, 24–28]. BIM deletion polymorphism is an independent predictive factor of response to EGRF TKIs. Patients with a BIM del+ have low response rate to EGFR TKIs and have inferior clinical outcomes (PFS and or OS) compared to patients without BIM deletion [20, 25, 27]. BIM deletion polymorphism is relatively common in East Asians, but unusual in the European and African populations [20]. Our study documented for the first time the prevalence of BIM deletion polymorphism in the Latin American population (15.7 %; 14 of 89 patients). This prevalence is similar to that previously reported in the Asian population [24–26, 28]. We did not analyze the prevalence of BIM deletion polymorphism in healthy volunteers. In this study we also found that BIM deletion polymorphism was not related with any clinical or pathological factor and its prevalence is independent of the type of EGFR activating mutation.

Ng et al. showed that BIM deletion polymorphisms are associated with inferior clinical outcomes in patients with NSCLC who received EGFR TKIs therapy [20]. In Ng el al, study patients with BIM del+ had a shorter PFS (6.6 moths) compared with BIM del- patients (11.9 months) (n = 141, p = 0.0027). Other studies from the Asian population have shown similar results demonstrating that the presence of BIM deletion polymorphism is a negative predictive factor of response rate, PFS and OS to EGFR TKIs [24, 25, 27]. In a meta-analysis of six original eligible studies including 871 NSCLC patients [29], patients BIM del+ had poor response to EGFR TKI therapy (p= 0.001, OR = 0.39; 95% CI = 0.23–0.67). Disease control rate (DCR) with EGFR TKI treatment was significantly decreased in BIM del+ patients (p= 0.007, OR = 0.46, 95% CI = 0.25–0.85). Also, PFS and OS were significantly shorter in NSCLC EGFR-mutated patients with BIM deletion polymorphism (PFS: p< 0.001, HR = 1.37, 95% CI = 1.09–1.71; OS: p = 0.003, HR = 1.25, 95% CI = 1.08–1.45). Our results are consistent with these studies, suggesting that NSCLC EGFR mutation positive patients with BIM deletion polymorphism benefit less from EGFR TKI therapy in terms of PFS and OS compared to patients without BIM deletion polymorphism. BIM deletion polymorphism was an independent indicator of shorter PFS and OS in our population.

In the literature there are other studies with contradictory results to our study, failing to demonstrate an association between BIM deletion polymorphism and the response to EGFR TKI therapy [26, 28]. For example, Lee et al analyzed the influence of BIM deletion polymorphism in 205 NSCLC EGFR mutation positive patients [28]. BIM del+ patients had similar objective response rates compared to BIM del- patients (91% vs. 84%, p = 0.585). PFS and OS did not differ significantly between both molecular selected populations (PFS = 12 vs. 11 months, p = 0.160; OS = 31 vs. 30 months, p = 0.452). Similar results were reported in another study performed in the Asian population [26]. Different hypothesis have been proposed to explain these contradictory results. For instance, the response to EGFR TKIs varies according to the level of the proapoptotic Bcl-2-homology domain 3 (BH3). Such changes in BH3 and not only the presence of BIM polymorphism itself could therefore explain these diverging results [30]. Likewise, there may be additional ethnic differences in BIM polymorphisms between East Asian and Latin American. Therefore measuring BIM mRNA levels before treatment should be encouraged to establish the role of BIM as a predictor of response to EGFR TKI therapy [30, 31].

Other pro-apoptic proteins belonging to BCL-2 family such a BAX, BAK, PUMA and BAD might also play an important role in the response in oncogene-addicted cancer and activation of apoptosis in NSCLC [32–35]. Variations of the expression of these BCL-2 family proteins could influence the response to TKI therapy in the studies where BIM polymorphisms were evaluated. Further examination of additional genes such as TP53, PTEN and PIK3CA mutations might be useful to unveil the variety of responses to EGFR TKIs [34, 36, 37].

The present study had several limitations including sample size and bias related to the retrospective nature of data collection. We did not analyze BIM deletion polymorphism in blood samples, however there seems to be a concordance between peripheral venous blood and FFPE [25]; still, the validation of BIM deletion polymorphisms in blood samples is warranted as a non-invasive method that allows tissue sparing. Also, we did not validate other genetic alterations such a BCL-2 family proteins distinct to BIM, PTEN, PI3K, etc., in order to explain different responses to EGFR TKIs.

## MATERIALS AND METHODS

### Patients and samples

This is a retrospective study following the results described by Ng and colleagues [20]. We included 89 patients carriers of EGFR mutations evaluated at the Clinical and Applied Cancer Research Foundation in Bogotá, Colombia. Samples and information were collected from January 1, 2011 to March 31, 2014. All patients met the following inclusion criteria: informed consent; histological confirmed non-squamous NSCLC, locally advanced or advanced disease (stage IV), no previous systemic treatment, age >18 years; and adequate formalin-fixed, paraffin-embedded (FFPE) tissue available to detect EGFR mutations and their BIM polymorphism status. We also obtained a complete medical history, laboratory tests results, and radiology examinations for each patient. All cases were treated with erlotinib 150 mg daily until disease progression or intolerable toxicity.

### DNA extraction and EGFR mutation detection

DNA from tumor tissue was extracted using the DNeasy Tissue Kit or the QIAamp DNA FFPE Tissue Kit (Qiagen, Hilden, Germany) according to the manufacturer's protocol. EGFR mutations were studied by COBAS 8100 (Cobas real-time PCR platform, Roche Diagnostics, Indianapolis, Indiana, US).

### BIM genotyping and direct sequencing

All samples were amplified by polymerase chain reaction (PCR) to detect *BIM* polymorphisms using the following primer sequences: wild-type (WT) *BIM* forward primer, 50-ACTGTAAAACGACGGCCAGTCCTCATGATGAAGGCTAACTCAA-30; and reverse primer, 50-ACCAGGAAACAGCTATGACCAACCTCTGACAAGTGACCACCA-30. For the BIM deletion polymorphism, the forward primer sequence was the same as that used for wild type BIM, and the reverse sequence was 50-ACCAGGAAACAGCTATGACCGGCACAGCCTCTATGGAGAACA-30. The reaction condition was 95°C for 10 minutes followed by 40 cycles at 94°C for 30 seconds, 60°C for 30 seconds, and 72°C for 30 seconds; and a final extension at 72°C for 10 minutes using the Taq Polymerase premix PCR Kit (Applied Biosystems). PCR products (177 base pairs [bp] for the BIM deletion polymorphism and 174 bp for wild-type BIM were then separated on a 3% agarose gel with nucleic acid dye by electrophoresis and were purified before direct sequencing. To check the presence of somatic mutations in the BCL2L11 gene, a comprehensive screening was performed by direct sequencing including rare mutations described in COSMIC (0.2%; p.Q37Q, p.G49R, p.R85I, p.F97L, p.R188L, p.W195C) without finding any.

### Statistical analysis

Statistical analyses were conducted using SPSS software 19.0 (SPSS, Chicago, IL, U.S.A.). Differences in clinical characteristics, overall response rate (ORR), PFS, OS and adverse events of patients with or without BIM deletion polymorphism (BIM*-del*+; *BIM del-*) were compared using the Pearson chisquare test or the Fisher's exact test. Survival curves were drawn by the Kaplan–Meier method, and statistical analysis was performed using the log-rank test. We used univariate analysis and multivariate Cox regression analysis (including type of EGFR mutation, BIM-*del*, response to TKIs, ECOG and brain metastases) to identify factors associated with PFS and OS. We studied the following clinical characteristics: age, sex, performance status, stage, weight loss, site of metastasis (brain, bone, lung, liver, lymph nodes), type of EGFR mutation [common mutations (L858R and exon 19 deletion) vs. uncommon mutations], EGFR-TKI response, chemotherapy response, smoking history, and BIM*-del*. For any purpose ORR was defined as the proportion of patients with tumor size reduction during TKI treatment, PFS was defined as the length of time between starting TKI and disease progression or death, and OS is the period of time from date of diagnosis until death.

## CONCLUSIONS

The BIM deletion polymorphism is present in this Hispanic NSCLC EGFR mutated cohort of patients with a similar incidence to Asian countries. In our population, the presence of the BIM deletion polymorphism was an important and independent predictive factor of response when patients were treated with an EGFR TKI therapy.
